# Vancomycin Revisited – 60 Years Later

**DOI:** 10.3389/fpubh.2014.00217

**Published:** 2014-10-31

**Authors:** Ethan Rubinstein, Yoav Keynan

**Affiliations:** ^1^Department of Internal Medicine, University of Manitoba, Winnipeg, MB, Canada

**Keywords:** vancomycin, MRSA, VRE, “red man” syndrome, nephrotoxicity, oral-vancomycin, safety, dose

## Abstract

Vancomycin is one of the older antibiotics that has been now in clinical use close to 60 years. Earlier on, vancomycin was associated with many side effects including vestibular and renal, most likely due to impurities contained in early vancomycin lots. Over the years, the impurities have been removed and the compound has now far less vestibular adverse effects, but still possesses renal toxicity if administered at higher doses rendering trough serum levels of >15 mcg/mL or if administered for prolonged periods of time. Vancomycin is effective against most Gram-positive cocci and bacilli with the exception of rare organisms as well as enterococci that became vancomycin resistant, mostly *Enterococcus faecium*. The major use of vancomycin today is for infections caused by methicillin-resistant *Staphylococcus aureus* (MRSA), methicillin-resistant *Staphylococcus epidermidis* (MRSE) and amoxicillin-resistant enterococci. In its oral form, vancomycin is used to treat diarrhea caused by *Clsotridium difficile*. With *S. aureus*, there are only a handful of vancomycin-resistant strains. Nevertheless, a “vancomycin creep” that is slow upward trending of vancomycin MIC from <1 mcg/mL to higher values has been noted in several parts of the world, but not globally, and strains that have MIC’s of 1.5–2 mcg/mL are associated with high therapeutic failure rates. This phenomenon has also been recently recognized in methicillin-susceptible *S. aureus* (MSSA). While vancomycin is relatively a safe agent adverse events include the “red man” syndrome, allergic reactions, and various bone marrow effects as well as nephrotoxicity. Vancomycin has been a very important tool in our therapeutic armamentarium that remained effective for many years, it is likely remain effective as long as resistance to vancomycin remains controlled.

Vancomycin was isolated in 1957 by Dr. E.C Kornfield, an organic chemist with Eli Lilly in the deep jungles in Borneo from a fungus named *Streptomyces orientalis*. Soil samples from that jungle of where the fungus was isolated yielded in broth fermentation a compound that was highly effective and bactericidal against *Staphylococci*. The initial compound was labeled 05865 and initial studies have shown that *Staphylococci* failed to develop resistance to 05865 following serial passages in media containing this agent. As in this period, there was a growing problem of drug-resistant *Staphylococci*, the FDA granted 05865 a “fast track approval,” on the basis of open – labeled studies submitted to the agency in 1958. 05865 was subsequently labeled as “vancomycin,” a term derived from the word vanquish. The original product-vancomycin, obtained by fermentation, contained considerable amounts (up to 70%) of impurities, and had a brown color earning it the nickname “Mississippi Mud” (1, 2).

Vancomycin is active against Gram-positive aerobic cocci and bacilli, e.g., *Staphylococci, Streptococci, Enterococci*, and *Pneumococci* as well as *Corynebacterium, Listeria, Bacillus spp, Clostridia*, and oral Gram-positive anaerobes. Vancomycin is active against methicillin-resistant *Staphylococcus aureus* (MRSA) and against methicillin-resistant *Staphylococcus epidermidis* (MRSE), as well as against penicillin-resistant *Corynebacterium jeikeium, Streptococcus pneumoniae*, and *Clostridium difficile. S*trains of *Leuconosoc, Lactobacillus, Pediococcus*, and *Erysipelothrix* possess inherent resistance to vancomycin.

Vancomycin remains the first-line agent for methicillin-resistant coagulase-negative and coagulase-positive staphylococcal infections, including bacteremia, endocarditis, pneumonia, cellulitis, and osteomyelitis. In addition, it is used to treat serious Gram-positive infections among patients who are allergic to semi-synthetic penicillins or cephalosporins (3).

While vancomycin is bactericidal against all susceptible Gram-positive pathogens it exerts only bacteriostatic activity against enterococci and needs to be combined with another agent, usually an aminoglycoside, to achieve bactericidal activity. In addition, vancomycin possesses activity against Gram-positive anaerobes including *Clostridium* species as well as oral anaerobes such as peptostreptococcus, propiobacterium, etc. (including *C. difficile*), but is lacking activity against Gram-negative bacilli. Vancomycin “slow bactericidal” activity against MSSA, compared to β-lactams, this slow activity is reflected also in the worse clinical outcomes of cases of MSSA bacteremia and pneumonia treated with vancomycin (4–7).

Vancomycin is a tricyclic glycopeptide (Figure 1) that consists of seven membered peptide chains forming the tricyclic structure and attached disaccharide composed of vancosamine and glucose. The molecular weight is 1485 and thus vancomycin is heavier that most β-lactam antibiotics, but similar in weight to the unrelated compound daptomycin. It has similar molecular weight to teicoplanin and its derivative dalbavancin, to the semi-synthetic telavancin, as well as the more distantly related semi-synthetic lipoglycopeptide oritavancin (8).

**Figure 1:**
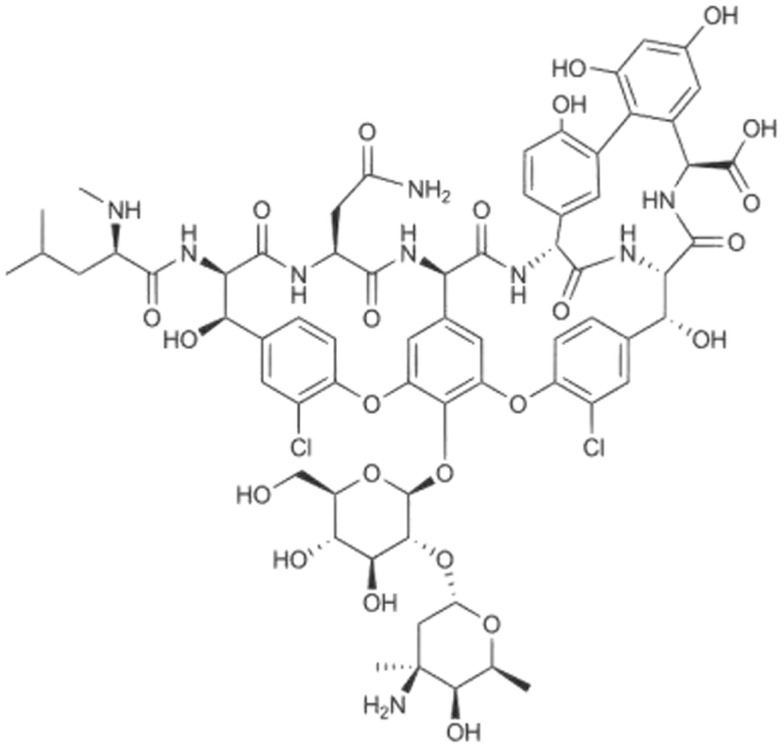
**Vancomycin structure**.

Vancomycin and the above mentioned compounds inhibit cell wall synthesis in its later stages thus affecting dividing bacteria. The target of their activity is the murein monomers, which are peptidoglycan precursors. The murein monomers are added to the peptidoglycan by transglycosylation followed by transpeptidation. Vancomycin binds to the d-ala-d-ala moiety of the monomers, subsequently the monomers cross the cell membrane. This complex leads to conformational change that blocks the glycosyltransferase leading to inhibition of the incorporation of the murein monomers to the growing peptidoglycan chain and preventing further transpeptidation with consequent interruption of the cell wall synthesis.

When methicillin and subsequently other anti-staphylococcal penicillins (cloxacillin, dicloxacillin, flucloxacillin, etc.) were introduced to the market, the clinical utility of vancomycin was displaced from its position as first-line anti-staphylococcal therapy to second-line anti-staphylococcal therapy, mainly because of its nephrotoxicity and ototoxicity observed in the early clinical trials (1). It is clear today, that earlier observations of nephrotoxicity and ototoxicity were largely due to the impurities of the earlier preparations. The additional compounds accounted to up to 70% of vancomycin contained in the injection vials. Later, in the 1970s when newer preparations of vancomycin were introduced, the issue of ototoxicity has essentially disappeared and nephrotoxicity was diminished considerably unless vancomycin was used in combination with aminoglycosides; the same was also seen in animal experiments (9, 10).

Vancomycin is in broad clinical use with the main indications including skin and soft tissue infections and osteomyelitis. It is used for these indications empirically, prior to availability of culture results, and when MRSA is the culprit. Similarly, it is frequently used for treatment of bacteremia and endocarditis where MRSA is deemed a possible cause. In this context, once MSSA is documented as the causative agent, vancomycin has been associated with inferior outcomes and should therefore not be used for MSSA bacteremia or endocarditis (4–7). In addition, vancomycin is used to treat gram-positive pneumonia, primarily in context of hospital-acquired infections and in the treatment of bacterial meningitis caused by penicillin-resistant *Streptococcus pneumonia*. The worldwide emergence of methicillin-resistant *Staphylococci* (MRSA and MRSE) in the 1970s, brought vancomycin back to the stage. At this juncture, pharmacokinetics became popular and blood concentrations were determined, and the first normograms with dosage adjustments for patients with renal impairment were published (11).

The subsequent emergence of vancomycin resistance among enterococci in the mid-1980s and failure of such patients even if they had enterococci with vancomycin-intermediate susceptibility was described, resulting in vancomycin losing it omnipotence for all gram-positive cocci (12). The appearance of *S. aureus* strains with intermediate susceptibility to vancomycin (VISA) and vancomycin-resistant *S. aureus* (VRSA) (12–14) and vancomycin-resistant *S. epidermidis* (VRSE) (15, 16), brought an end to the hitherto hegemony of vancomycin in the Gram-positive coccal arena. Fortunately, the occurrence of these difficult to treat *Staphylococci* remains rare.

Normally, the suggested minimal inhibitory concentration (MIC) of vancomycin against *S. aureus* was ≤2 mcg/mL. It has been repeatedly shown that clinical failures in patients with endocarditis, bacteremia, and pneumonia occur when the *S. aureus* strains causing these infections have MIC of ≥2 mcg/mL (17–19). Even within the range of MICs below 2 mcg/mL, a high failure rate was also observed in patients infected with strains that has vancomycin MIC of 1.5 (20, 21).

A phenomenon named “vancomycin Creep” was described in recent years denoting a slow but steady increase in vancomycin MIC observed over time, from values of 0.5–0.75 mcg/mL to levels of 1.25–1.5 mcg/mL (22, 23). In some parts of the world, however, this phenomenon of “vancomycin creep” does not occur (24).

Therapeutic monitoring of vancomycin levels in adults: for many years a disagreement existed regarding the need for measurement of vancomycin levels. The discussion was justified as long as most vancomycin MIC were ≤1 mcg/mL, this situation has, however, changed and with increasing MIC’s a decreased clinical success became evident (25, 26). In patients infected with strains that had MIC of 4 mcg/mL and above the failure rate of vancomycin reached 60%, this has led many bodies (FDA, ICLS) to reduce in 2008 the limit of susceptibility (breakpoint) to 2 mcg/mL. The other reason for measuring serum concentrations is to avoid toxicity, which becomes apparent when serum trough levels exceed 15 mcg/mL (27–29).

Vancomycin resistance among enterococci is attributed to change in the d-alanyl-d-alanine portion of peptide precursor units, transmitted as Van genes, thus rendering it incapable of inhibiting peptidoglycan polymerase and transpeptidation reactions. While the mechanisms that lead to vancomycin resistance are clear, the reasons behind the emergence of these strains are attributed to a myriad of causes (30–32). Thus far, at least eight types of acquired resistance to glycopeptides have been reported on the basis of phenotypic and genotypic criteria (VanA, VanB, VanD, VanE, VanG, VanL, VanM, VanN, and VanC). VanC being constitutively expressed by *Enterococcus gallinarum* and *Enterococcus casseliflavus* (33, 34). Carriage of VRE in the gastrointestinal tract occurs more commonly after prolonged hospital admissions and more frequently in high-risk units, such as intensive care units (ICUs), hemato-oncology, and abdominal surgery wards. VRE colonization may persist for years, and a recent study from Korea identified vancomycin exposure after colonization as the strongest predictor of prolonged colonization, with odds ratio of 4.05 in a multivariate model adjusting for potential confounders (35). It has been speculated that exposure to oral vancomycin is an important contributor to propagation of VRE in the healthcare setting, as it drives prolonged colonization with VRE from a myriad of sources (36, 37).

The challenge posed by MRSA with increasing vancomycin MIC’s has led to attempts to treat such patients with higher doses of vancomycin aiming to attain a trough level of >15 mcg/mL, generally these attempts have not been associated with increased success rate, but rather with increased nephrotoxicity (38, 39). These doses are used in the context of *S. aureus* infections.

The effect of vancomycin antibacterial activity is pharmacokinetically dependent on the time that the serum concentration of vancomycin is above the MIC. *In vitro* and the neutropenic mouse thigh infection models have demonstrated that the area under the concentration curve (AUC) divided by the MIC (AUC/MIC) is the best predictor of the activity of vancomycin against methicillin-susceptible *S. aureus* (MRSA), and glycopeptide-intermediate *S. aureus* (GISA) (40). However, in a *Streptococcus pneumoniae* non-neutropenic mouse peritonitis model, Knudsen et al. (41) demonstrated that the peak serum concentration divided by the MIC (peak/MIC) was the pharmacodynamic parameter with the most predictive value. In patients with MRSA pneumonia an AUC/MIC value of ≥400 was associated with a successful outcome, whereas an AUC/MIC value of ≤400 was associated with a lower eradication rate and a higher mortality rate (*P* = 0.005) (42) With strains that have a MIC of ≥2 mcg/mL this desired AUC/MIC ratio of 400 is more difficult to achieve and attainment of lower AUC/MIC ratios has been associated with poorer outcomes in the context of bacteremia (43, 44).

Vancomycin is not appreciably absorbed orally, and is eliminated primarily via the renal route, with >80–90% recovered unchanged in the urine within 24 h after administration of a single dose. The pharmacokinetic profile of vancomycin can be characterized by either a 2- or 3-compartment pharmacokinetic profile (45).

Vancomycin is normally administered intravenously, with a standard infusion time of at least 1 h′, to minimize infusion-related adverse effects. In patients with normal creatinine clearances, vancomycin has a α-distribution phase of ~30 min to 1 h′ and a β-elimination half-life of 6–12 h. The volume of distribution is 0.4–1 L/kg. The binding of vancomycin to protein ranges from 10 to 50%. Factors that affect the overall activity of vancomycin include its tissue distribution, inoculum size, and protein-binding effects (45–48).

Oral vancomycin is not absorbed systemically and achieves high levels in the colon thus oral vancomycin formulation may be used at a dose of 125 mg q6h, and less frequently a higher dose of 500 mg four times daily is equally effective (against *C. difficile* colitis). Oral vancomycin is indicated for patients with either severe *C. difficile* colitis (high leukocyte count >15,000, serum creatinine increase ≥50% from baseline), or those with *C. difficile* colitis that failed other therapeutic modalities (mainly metronidazole) (49). Intravenous vancomycin on the other hand, has no effect on *C. difficile* colitis since it is not excreted appreciably into the gastrointestinal tract. Intracolonic vancomycin administration at the same dose and frequency may be considered in patients with profound ileus. In patients with ileostomy, the same dose is administered as well.

Vancomycin-associated nephrotoxicity can still be seen, even in the presence of appropriate serum concentrations, especially when it is co-administered with aminoglycosides, amphotericin B, foscarnet, pentamidine ACE inhibitors, loop diuretics, cyclosporine, cyclophosphamide, and the platinums but also when vancomycin therapy exceeds 14 days and in patients with very high APACHE scores (50).

The most common adverse events that are not related to vancomycin serum levels are: fever, chills, and phlebitis. The “red man” (Figure 2) syndrome (RMS) is manifested by: tingling and red flushing of the face, upper torso, and upper extremities. It is thought to be mediated by histamine release from mast cells, it is considered as pseudo-allergic drug reaction without underlying immunological processes (51). The phenomena is enhanced by co-administration of opiates (52) and can be diminished or aborted by slowing the infusion rate of vancomycin to ≤10 mg/min, and premedication with diphenhydramine (50 mg orally or intravenously) as well as ranitidine (50 mg orally or intravenously) (53). Should higher doses of vancomycin be administered (>1 g′) the infusion time needs to be prolonged if the RMS occurs.

**Figure 2:**
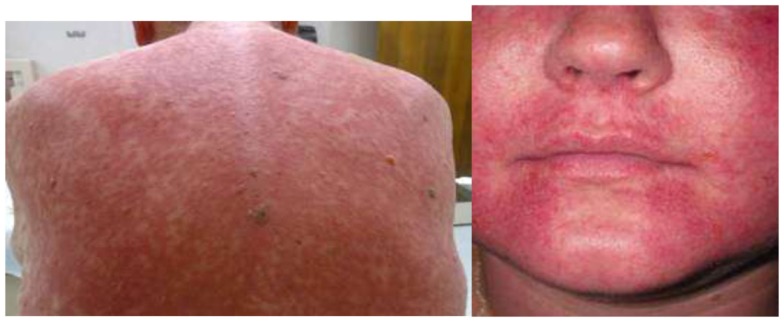
**“Red man” syndrome**.

Neutropenia is observed not infrequently in patients receiving vancomycin for longer periods of time. The exact incidence is difficult to ascertain due to co-administration of other potentially marrow suppressive medications and due to the underlying sepsis or diseases. This adverse event is not related to vancomycin serum concentrations, and is reversible when the agent is discontinued (54).

Thrombocytopenia (immune), as well as leukocytosis, eosinophilia, and leukocytoclastic vasculitis have been associated with vancomycin (55, 56). Drug fever is considered infrequent and occasionally appears along with neutropenia (57).

Anaphylactic reaction is mediated by drug-specific IgE antibodies. Patients with anaphylactic reactions to vancomycin often have a history of multiple prior exposures, the reaction is considered rare, although angioedema, respiratory distress, and bronchospasm, with demonstrable drug-specific IgE have been described (58).

A cross allergic reaction between vancomycin and other glycopeptides has been described for teicoplanin but not for other glycopeptides (59).

Other dermatological manifestation of vancomycin are: DRESS (drug rash with eosinophilia and systemic symptoms) also named DiHS (drug-induced hypersensitivity syndrome). This is a systemic response, in which rash, mucosal involvement, atypical lymphocytes, frequent eosinophilia, and lymphadenopathy occur together with organ involvement (kidneys, liver, myocarditis/pericarditis). Treatment includes discontinuation of drug therapy and avoiding its use in the future and glucocorticoids for a short duration (60–62).

Vancomycin-related linear IgA bullous dermatosis (LABD) is a rare, autoantibody-mediated skin reaction. LABD may be confused with toxic epidermal necrolysis. The blistering bullous lesions of LABD need to be differentiated from pemphigoid, erythema multiforme, and dermatitis herpetiformis. Direct immunofluorescence is usually needed to confirm the diagnosis of LABD. Linear IgA deposition at the dermal–epidermal junction of the basement membrane zone is the characteristic finding of LABD. LABD can appear suddenly, appears to be idiosyncratic and unrelated to serum vancomycin levels (63, 64). Vancomycin is the most common cause of drug-induced LABD (65).

Other severe cutaneous syndromes including Stevens–Johnson syndrome, exfoliative dermatitis, toxic epidermal necrolysis, extensive fixed drug eruption, and leukocytoclastic vasculitis have all been described in association with vancomycin use (66, 67).

Desensitization procedure may be indicated for suspected IgE-mediated reactions and for severe RMS that is refractory to other measures and is less common with availability of appropriate alternatives (such as linezolid, daptomycin, telavancin, quinupristin/dalfoprisin, tigecycline, and ceftaroline-all active against MRSA). Desensitization is contraindicated in patients with the DRESS syndrome and in those with exfoliative skin reactions such as Stevens–Johnson syndrome and toxic epidermal necrolysis. Desensitization for IgE-mediated allergy is usually performed in an intensive care unit. Desensitization should be performed immediately before the required treatment, because maintaining tolerance requires continual exposure to the drug. A rapid desensitization protocol (after up to date) is shown in Table 1.

**Table 1:** **Rapid vancomycin desensitization protocole (after up to date)**.

**Premedication:**			
Diphenhydramine 50 mg IV and hydrocortisone 100 mg IV 15 min prior to initiation of protocol, then every 6 h throughout protocol			

**Infusion no**.	**Dilution**	**Vancomycin dose (mg)**	**Concentration (mg/mL)**

1	1:10,000	0.02	0.0002
2	1:1000	0.2	0.002
3	1:100	2	0.02
4	1:10	20	0.2
5	Standard	500	2

**Preparation**
1. Prepare a standard bag of 500 mg vancomycin in 250 mL NS or D5W; label as infusion number 5, vancomycin 2 mg/mL.			
2. Draw up 10 mL of the standard vancomycin 2 mg/mL preparation and place in 100 mL bag of NS or D5W; label as infusion number 4, vancomycin 0.2 mg/mL.			
3. Draw up 10 mL of the 0.2-mg/mL solution and place in a 100-mL bag of NS or D5W; label as infusion number 3, vancomycin 0.02 mg/mL.			
4. Draw up 10 mL of the 0.02-mg/mL solution and place in a 100-mL bag of NS or D5W; label as infusion number 2, vancomycin 0.002 mg/mL.			
5. Draw up 10 mL of the 0.002-mg/mL solution and place in a 100-mL bag of NS or D5W; label as infusion number 1, vancomycin 0.0002 mg/mL.			
**Infusion rate directions**			
Initiate infusion rate at 0.5 mL/min (30 mL/h) and increase by 0.5 mL/min (30 mL/h) as tolerated every 5 min to a maximum rate of 5 mL/min (300 mL/h). If pruritus, hypotension, rash, or difficulty breathing occurs, stop infusion and reinfuse the previously tolerated infusion at the highest tolerated rate. This step may be repeated up to three times for any given concentration.			
Upon completion of infusion number 5, immediately administer the required dose of vancomycin in the usual dilution of NS or D5W over 2 h. Decrease rate if patient becomes symptomatic or, alternatively, increase rate if patient tolerated dose. Administer diphenhydramine 50 mg orally 60 min prior to each dose.			

Mild symptoms may occur in 20–30% during desensitization and are usually self-limited (e.g., flushing, pruritus, limited urticaria), and can be managed without discontinuation of the desensitization protocol (68). Mild symptoms are managed interruption of infusion and treating the symptoms that do not subside spontaneously. Once symptoms have subsided, the last tolerated step is repeated. If moderate or severe symptoms develop, the infusion should be discontinued and the symptoms treated. If vancomycin is discontinued, even for a short while, desensitization needs to be repeated in the previously described manner as the chances for anaphylaxis following the re-start of the vancomycin therapy are considerable (69).

Vancomycin has been a valuable agent for the management of infections caused by gram-positive bacteria for many decades. Attempts produce related derivatives as well as related compounds with similar or better antimicrobial spectrum, particularly against vancomycin-resistant enterococci and strains of VISA and MRSA with increased vancomycin MIC’s are ongoing with notable examples including telavancin, dalbavancin, and oritavancin, the latter two poses longer half-lives and can be administered once-weekly. In addition distinct classes with similar spectrum of activity such as daptomycin and the streptogramin quinupristin/dalfopristin may be used.

In clinical trials, these compounds were compared to vancomycin and have been shown to be non-inferior (38, 70–76). This leads us to believe that vancomycin will continue to be used as the major glycopeptide antibiotic against MRSA and enterococci that are vancomycin susceptible, as long as agents with superior performance are not available.

## Conflict of Interest Statement

The authors declare that the research was conducted in the absence of any commercial or financial relationships that could be construed as a potential conflict of interest.
